# The plant defense and pathogen counterdefense mediated by *Hevea brasiliensis* serine protease *HbSPA* and *Phytophthora palmivora* extracellular protease inhibitor *PpEPI10*

**DOI:** 10.1371/journal.pone.0175795

**Published:** 2017-05-01

**Authors:** Kitiya Ekchaweng, Edouard Evangelisti, Sebastian Schornack, Miaoying Tian, Nunta Churngchow

**Affiliations:** 1 Department of Biochemistry, Faculty of Science, Prince of Songkla University, Hat Yai, Songkhla, Thailand; 2 Department of Plant and Environmental Protection Sciences, University of Hawaii at Manoa, Honolulu, Hawaii, United States of America; 3 East-West Center, Honolulu, Hawaii, United States of America; 4 Sainsbury Laboratory Cambridge University, Cambridge, United Kingdom; Pennsylvania State University, UNITED STATES

## Abstract

Rubber tree (*Hevea brasiliensis* Muell. Arg) is an important economic crop in Thailand. Leaf fall and black stripe diseases caused by the aggressive oomycete pathogen *Phytophthora palmivora*, cause deleterious damage on rubber tree growth leading to decrease of latex production. To gain insights into the molecular function of *H*. *brasiliensis* subtilisin-like serine proteases, the *HbSPA*, *HbSPB*, and *HbSPC* genes were transiently expressed in *Nicotiana benthamiana* via agroinfiltration. A functional protease encoded by *HbSPA* was successfully expressed in the apoplast of *N*. *benthamiana* leaves. Transient expression of *HbSPA* in *N*. *benthamiana* leaves enhanced resistance to *P*. *palmivora*, suggesting that HbSPA plays an important role in plant defense. *P*. *palmivora* Kazal-like extracellular protease inhibitor 10 (PpEPI10), an apoplastic effector, has been implicated in pathogenicity through the suppression of *H*. *brasiliensis* protease. Semi-quantitative RT-PCR revealed that the *PpEPI10* gene was significantly up-regulated during colonization of rubber tree by *P*. *palmivora*. Concurrently, the *HbSPA* gene was highly expressed during infection. To investigate a possible interaction between HbSPA and PpEPI10, the recombinant PpEPI10 protein (rPpEPI10) was expressed in *Escherichia coli* and purified using affinity chromatography. In-gel zymogram and co-immunoprecipitation (co-IP) assays demonstrated that rPpEPI10 specifically inhibited and interacted with HbSPA. The targeting of HbSPA by PpEPI10 revealed a defense-counterdefense mechanism, which is mediated by plant protease and pathogen protease inhibitor, in *H*. *brasiliensis*-*P*. *palmivora* interactions.

## Introduction

Rubber tree (*Hevea brasiliensis* Muell. Arg) is the major source for natural latex production and one of economically important crops in Thailand. *Phytophthora palmivora* is known as an aggressive oomycete pathogen of many woody plants and food crops worldwide [1]. It attacks the petioles, leaf, root or tapping panel of the rubber tree, causing severe damages on plant growth and crop productivity, resulting in enormous economic loss [2–3]. Although this pathogen has been considered as a disastrous pathogen, molecular mechanisms involved in pathogenicity are largely unknown. An understanding of the molecular basis of host-pathogen interactions is expected to provide significant knowledge leading to the development of effective disease control measures.

To promote colonization, *Phytophthora* species interfere with host innate immune systems by secreting hundreds of effector proteins to manipulate host cell physiology, thereby facilitating infection and reproduction [4–5]. Some effector proteins possess biochemical activities that counteract their host targets to suppress primary defenses, such as *P*. *infestans* Kazal-like extracellular protease inhibitors EPI1 and EPI10 [6–7] and *P*. *sojae* glucanase inhibitor protein1 (GIP1) [8].

To successfully overcome pathogen invasion, plants develop effective defense strategies against challenging pathogens. One of the strongest plant defensive arsenals is the synthesis of pathogenesis-related (PR) proteins, which accumulate locally in the primarily infected and surrounding tissues, and also in systemic tissues [9]. So far at least 17 families of PR proteins are recognized according to their properties and functions [10]. Some of them, such as PR2 (β-1, 3-glucanases), PR3 (chitinases) and PR7 (endoproteases), might be directly antimicrobial, by catalyzing the degradation of structural components in pathogen cell walls. These hydrolytic enzymes are secreted to the plant extracellular matrix (intercellular space) where host-pathogen interaction, recognition, and signaling processes occur [10–11].

Subtilisin-like serine proteases or subtilases (EC 3.4.21.14) are serine endopeptidases containing a highly conserved catalytic triad motif that is comprised of aspartate, histidine, and serine [12]. They are ubiquitously found in plants and play essential roles in multiple physiological, morphological and biochemical processes, including responses to environmental stress [13]. The availability of the complete genomic sequence of some model species revealed that plant subtilases constitute a large gene family, comprising 56 genes in *Arabidopsis* (*Arabidopsis thaliana*), 63 genes in rice (*Oryza sativa*), 15 genes in tomato (*Lycopersicon esculentum*) and 80 genes in grape (*Vitis vinifera*) [14–17]. A more extensive expansion in the number of subtilase genes in plants compared to animals is accompanied by their diverse functions or various evolutionary mechanisms in plants [17].

The first evidence regarding the importance of plant subtilases in defense mechanisms was reported in tomato. P69B, an apoplastic subtilase known as a PR-protein (PR-7 class), is induced during infection by various plant pathogens, such as citrus exocortis viroid, *Pseudomonas syringe*, *P*. *infestans*, and salicylic acid treatment [6,18–21]. In addition, P69C, another isoform of the P69 subtilase family, is also induced by pathogen infection and is hypothesized to participate in pathogen perception and initiation of signaling cascades by specific processing of a LRP (leucine-rich repeat protein) in infected tomato plants [22]. Besides, the *Arabidopsis SBT3*.*3* gene, encoding an extracellular subtilase homologous to the tomato P69C, is rapidly up-regulated in response to *Pseudomonas syringae* and implicated in the establishment of immune priming by induction of SA-responsive genes [23]. Moreover, the overexpression of an extracellular *subtilase* gene (*GbSBT1*) from *Gossypium babardense* enhances the transcripts of defense-related genes and the disease resistance of *Arabidopsis* to *Fusarium oxysporum* and *Verticillium dahliae* [24].

Interestingly, a critical role of subtilases in plant immunity is also implied by the observation that many pathogens produce various effectors that target these proteases. Examples are *P*. *infestans* EPI1 and EPI10, two Kazal-like extracellular serine protease inhibitors that are identified to specifically inhibit and bind to tomato P69B. Inhibition of P69B by two distinct and structurally divergent protease inhibitors is consistent with the hypothesis that P69B is potentially harmful to this pathogen and these pathogen-derived effectors function in counterdefense [6–7].

Furthermore, a Kazal-like extracellular serine protease inhibitor PpEPI10, a homolog of EPI10 from *P*. *infestans*, has been reported to be secreted by *P*. *palmivora*. Two Kazal domains (Kazal1 and Kazal2) of PpEPI10 inhibit and interact with a 95 kDa protease from rubber tree leaf extracts, suggesting a virulence role of PpEPI10 in pathogenicity [25]. However, the functional activity of PpEPI10 in suppressing immune responses in rubber tree remains largely unexplored. In addition, the 95kDa protease was not identified previously. The present study functionally characterized the biochemical activity of a novel subtilisin-like serine protease from *H*. *brasiliensis* (HbSPA) and its defensive role in plants against *P*. *palmivora* though *Agrobacterium*-mediated transient expression in *N*. *benthamiana*. *P*. *palmivora* PpEPI10 was found to inhibit and interact with HbSPA using in-gel zymogram and co-immunoprecipitation assays, suggesting that one of the antagonistic interactions between rubber tree and *P*. *palmivora* is mediated by a plant defensive protease and a pathogen protease inhibitor involved in counterdefense.

## Materials and methods

### *Phytophthora palmivora* culture

*P*. *palmivora* PP3-3 isolated from a diseased papaya plant [26] was cultured on 10% unclarified V8 agar (10% V8 juice, 0.1% CaCO_3_, 1.5% agar) at 25°C. For zoospore preparation, *P*. *palmivora* was cultured under fluorescent light for 5 days. Ten milliliters of cold (4°C) sterile distilled water was added to the growing *P*. *palmivora* mycelium. The plate was incubated at 4°C for 15 min and further shaken at 50 rpm at room temperature for 15 min to release the motile zoospores from sporangia. The concentration of zoospores was determined using a haemocytometer under a light microscope.

For RNA extraction, 1-cm agar plugs of *P*. *palmivora* mycelium from a 7-day-old culture were inoculated in 150 x 25 mm petri-dishes containing 20 ml of Plich liquid medium [26] and grown at 25°C in darkness for 7 days. The mycelium mats were harvested by vacuum filtration of the culture through a double-layer filter paper and washed twice with sterile water. The harvested mycelium mats were allowed to dry under sterile condition, fast-frozen in liquid nitrogen and stored at -80°C until use.

### Plant growth and infection assay

Twenty-one-day-bud-grafted rubber seedlings (*H*. *brasiliensis*, RRIM600 cultivar) were propagated in a climate-controlled room at 25°C under 12h-light and 12h-dark cycle. For the time-course of *P*. *palmivora* colonization of rubber tree, the rubber leaves at the developmental B2C stage of plantlets with uniform growth were inoculated with 1 droplet (30-μL) containing ~3,000 zoospores of *P*. *palmivora* on the underside of the leaves. The treated rubber seedlings were further incubated in a climate-controlled room at 25°C, under 12h-light and 12h-dark cycle. The infected leaves were harvested at 0, 1, 2, 3, 4, and 5 days post-inoculation. Leaf discs of similar size (1.5 × 1.5 cm) were dissected around the inoculated regions with the inoculation spot at the center of the sampled area. The leaf samples were flash-frozen in liquid nitrogen and subsequently stored immediately to a -80°C freezer until RNA extraction.

*N*. *benthamiana* plants were grown in pots and maintained inside a growth chamber at 22°C, 60% humidity, under 16h-light and 8h-dark cycle. 6-week-old *N*. *benthamiana* plants were used for all agro-infiltration experiments.

### Total RNA extraction and first-strand cDNA synthesis

*P*. *palmivora* mycelium mats and rubber tree leaf samples were ground in liquid nitrogen to a fine powder with a mortar and pestle. Total RNA was isolated using the RNeasy^®^ Plant Mini Kit (Qiagen, CA, USA) according to the manufacturer’s instructions. The contaminating DNA was eliminated during total RNA extraction using an on-column RNase-free DNase I digestion set (Qiagen, CA, USA) according to the procedure of the RNeasy^®^ Plant Mini Kit. First-strand cDNA synthesis was performed using the Superscript^™^ III (Invitrogen, CA, USA). The obtained first-strand cDNAs were kept at -20°C until use.

### Expression pattern analysis of *PpEPI10* and *HbSPA*

To determine the *PpEPI10* gene expression during the infection of rubber tree leaves by *P*. *palmivora* using semi-quantitative reverse transcription polymerase chain reaction (semi-qRT-PCR), rubber tree leaves were inoculated with *P*. *palmivora* zoospores and total RNA was extracted from the infected leaves at 0, 1, 2, 3, 4, and 5 days post-inoculation as described previously. The specific primers, ExPpEPI10-F (5′CACCTAAATCGTCGGCAGACAACAGCTC3′), ExPpEPI10-R (5′ACCATCGTAATGTTTTTGCGCTCGTTGC3′), β-tubulin-F (5′GAAGTCATGTGCCTGGATAACGAG3′), and β-tubulin-R (5′CTCATGCGTCCGCGGAACATACAC3′) were designed based on the published *P*. *palmivora PpEPI10* (PLTG_2.01611) [27] and *β-tubulin* (GenBank accession no. JN203065) gene sequences and used for determining the expression of *PpEPI10* and *β-tubulin* transcripts, respectively. Amplification of *β-tubulin* gene was used as a constitutive control to determine the relative transcript level of *PpEPI10* gene. Two μL of first-strand cDNAs were used as a template in a 30-μL PCR reaction containing Emerald Amp^®^ GT PCR Master mix (Takara, Shiga, Japan), 0.2 μM of each gene-specific primer. The amplification was performed in a thermal cycler by preheating at 94°C for 5 min, followed by 30 cycles of denaturing at 94°C for 1 min, annealing at 60°C for 30 sec and extension at 72°C for 1 min, and a final extension step at 72°C for 10 min. The PCR products were analyzed by electrophoresis on a 1.2% (w/v) agarose gel, and visualized under the UV transilluminator and photographed by a Gel Document (UVP BioSpectrum^®^ MultiSpectral Imaging System^™^, Cambridge, UK).

To determine the *HbSPA* gene expression during infection of rubber tree leaves by *P*. *palmivora* using real-time quantitative reverse transcription polymerase chain reaction (real-time qRT-PCR), The specific primers, ExHbSPA-F (5′GCCACTGATTTTCTTCAAGAGTCAAACC3′) and ExHbSPA-R (5′TTGGATGGACAGTTGTATGGCTTATCAG3′), ExHbYLS8-F (5′CAAGCAAGAGTTCATTGACATTATTGAGACTG3′) and ExHbYLS8-R (5′ATCACAGGTTCTTACATAGTCGGATAGTC3′) were designed for determining the expression of the *H*. *brasiliensis subtilisin-like serine protease A* (*HbSPA*, GenBank accession no. KU845302) and the *H*. *brasiliensis mitosis protein YLS8* (*HbYLS8*, GenBank accession no. HQ323250), respectively. *HbYLS8* is stably expressed in the rubber tree [28] and used as an endogenous control. The real-time RT-qPCR was performed with 2 μL of first-strand cDNAs, gene-specific primers (final concentration of 0.9 μM for *HbSPA* and 1.0 μM for *HbYLS8*), and (2x) Luminaris Color HiGreen Low ROX qPCR Master Mix (Thermo Fisher Scientific, MA, USA) according to the manufacturer’s instructions on the Stratagene^™^ Mx3005P qPCR System (Agilent Technologies, CA, USA). The amplification results were automatically analyzed using the MxPro^™^ QPCR Software. Transcript abundance was calculated using the linearized plasmid DNA standard method as previously described [29]. The experiment was performed with three independent biological replicates.

### Bacterial strains and plasmids

*E*. *coli* DH5α (Invitrogen, CA, USA) or BL21 (DE3) (New England BioLabs, MA, USA) and *Agrobacterium tumefaciens* GV3101 [30] or C58-C1 [31] were used throughout this study and routinely maintained on Luria-Bertani (LB) agar or broth [32] at 37°C and 28°C, respectively. The pGD plasmid, a plant binary vector [33], was used to transiently express rubber tree serine proteases in *N*. *benthamiana*. The pJL3-*P19* plasmid was used to express *Tomato bushy stunt virus* P19 protein (GeneBank accession number M21958), a suppressor of post-transcriptional gene silencing to enhance the protein expression level in *N*. *benthamiana* [34–35].

### Plasmid construction and transient expression of *Hevea serine proteases* in *N*. *benthamiana*

The pGD-*HbSPA*, pGD-*HbSPB*, and pGD-*HbSPC* plasmids were constructed by cloning PCR-amplified DNA fragments corresponding to the open reading frames of *HbSPA*, *HbSPB*, and *HbSPC* fused with a hexahistidine(His)-tag encoding sequences at the C-terminus into the pGD vector [33]. The pJET-*HbSPA*, pJET-*HbSPB*, and pJET-*HbSPC* plasmids containing the open reading frames of *H*. *brasiliensis subtilisin-like serine protease A* (*HbSPA*), *H*. *brasiliensis subtilisin-like serine protease B* (*HbSPB*), and *H*. *brasiliensis subtilisin-like serine protease C* (*HbSPC*) (GenBank accession no. KU845302, KU845303, and KU845304, respectively) [29] were used as a template for PCR. Specific primers, HbSPA-F (5′GCGagatctATGAGGATCCGTAGCCATTCATG3′) and HbSPA-R (5′GCGgtcgacTTA*gtggtgatggtgatggtg*AGCTGAACCTGCATCTTTCTTCT3′), were used to amplify the open reading frame of *HbSPA* fragment fused with a hexahistidine(His)-tag encoding sequences at the C-terminus. The introduced *Bgl*II and *Sal*I restriction sites were underlined. The italic letters represent the His-tag sequence. Primers HbSPBC-F (5′GCGgtcgacATGGAAAACAGAAGATGCAAAAT3′) and HbSPBC-R (5′GCGggatccCTA*gtggtgatggtgatggtg*TTTAAATAAGACAGCAATTGGGC3′) were used to amplify the open reading frame of either *HbSPB* or *HbSPC* fused with a hexahistidine(His)-tag encoding sequences at the C-terminus. The introduced *Sal*I and *Bam*HI restriction sites were underlined. The His-tag sequence is shown in italic. The PCR was performed using the Phusion^®^High-Fidelity DNA polymerase (New England BioLabs, MA, USA) by preheating at 98°C for 30 s, followed by 35 cycles of denaturing 98°C for 15 s, annealing 60°C for 15 s and extension at 72°C for 2.3 min, and a final extension step at 72°C for 10 min. The amplified fragments were digested with appropriated restriction enzymes and cloned into the corresponding restriction sites of pGD vector [33]. The pGD-*HbSPA*, pGD-*HbSPB*, and pGD-*HbSPC* plasmids containing *HbSPA*, *HbSPB*, and *HbSPC* gene insert with the correct nucleotide sequences were used to transform *A*. *tumefaciens* stain C58-C1 by electroporation. To inhibit the post-transcriptional gene silencing (PTGS) that suppresses foreign gene expression in *N*. *benthamiana*, *A*. *tumefaciens* stain GV3101 containing pJL3-*P19* [34–35] was used in co-infiltration. Transient expression of *Hevea* serine protease genes in *N*. *benthamiana* using *A*. *tumefaciens* stain C58-C1 carrying pGD-*HbSPA*, pGD-*HbSPB*, or pGD-*HbSPC*, with or without *A*. *tumefaciens* stain GV3101 carrying pJL3-*P19*, was achieved by agroinfiltration following the methods previously described [36]. The expression of His-tagged HbSPA, HbSPB, and HbSPC proteins was analyzed by Western blot using HRP conjugated anti-His monoclonal antibody His-probe (H-3) (sc-8036HRP, Santa Cruz Biotechnology, INC., http://www.scbt.com) as described previously [36].

### Isolation of intercellular fluids

Intercellular (apoplastic) fluids were extracted from *N*. *benthamiana* leaves either infiltrated with *A*. *tumefaciens* stain GV3101 carrying pJL3-*P19* or a mixture of *A*. *tumefaciens* stain C58-C1 carrying pGD-*HbSPA* and *A*. *tumefaciens* stain GV3101 carrying pJL3-*P19* using a vacuum infiltration method [37]. Briefly, detached leaves were submerged in a cold extraction buffer (300 mM NaCl, 50 mM NaPO_4_, pH 7.0) [38] in a plastic container with the help of a plastic lined wire mesh and vacuum was applied in a Nalgene vacuum desiccator. Vacuum-infiltrated leaves were blot dried with soft paper towels to remove the liquid on the leaf surface, followed by centrifugation at 2,000 *g* for 20 min at 4°C to collect the intercellular fluids. The recovered fluids were filter-sterilized and analyzed by 10% SDS-PAGE and Western blot, or stored at -80°C.

### Plasmid construction, expression and purification of *PpEPI10*

Plasmid pET28a-*PpEPI10* was constructed by cloning a PCR-amplified DNA fragment corresponding to the mature protein of *PpEPI10* with the predicted signal peptide removed into *Bam*HI and *Sal*I sites of pET28a (Novogen). The specific primer pairs PpEPI10-F (5′GCGggatccATCGACGACGACAAGTGCTCA3′) and PpEPI10-R (5′GCGgtcgacTTACGACTGTTGCTCCTGCT3′) were designed based on the published *P*. *palmivora PpEPI10* sequence (PLTG_2.01611) [27]. The introduced *Bam*HI and *Sal*I restriction sites are underlined. The *PpEPI10* fragment was amplified by PCR using Phusion^®^High-Fidelity DNA polymerase (New England BioLabs, MA, USA) with *P*. *palmivora* mycelium cDNAs as the template. The resulting plasmid pET28a-*PpEPI10* was transformed into *E*. *coli* BL21 (DE3) cells by electroporation. The recombinant PpEPI10 protein expressed from pET28a-*PpEPI10* contains a His tag followed by a T7-tag at its N-terminus. Expression and purification of recombinant PpEPI10 proteins were performed as described previously [39]. The protein concentration was determined using Bradford protein assay kit (Bio-RAD, CA, USA).

### Protease activity and inhibition assay

The protease activity of HbSPA and its inhibition by PpEPI10 were determined by an in-gel protease assay using the Bio-Rad zymogram buffer system. Twenty microliters of the intercellular fluids containing the His-tagged HbSPA were incubated with or without various final concentrations (0.1, 0.4, 1.0, 4.0 μM) of the purified rPpEPI10 in a total volume of 22 μL for 30 min at 25°C and then mixed with equal volume of zymogram sample buffer (62.5 mM Tris-HCl, pH 6.8, 25% glycerol, 4% SDS, and 0.01% bromophenol blue). Ten microliters of the mixtures were loaded onto a 7.5% SDS-PAGE containing 0.1% gelatin in the absence of reducing reagent and boiling. After electrophoresis, the gel was incubated in 1x zymogram renaturation buffer (2.5% Triton X-100) for 30 min at room temperature and then equilibrated in 1x zymogram development buffer (50 mM Tris-HCl, pH 7.5, 200 mM NaCl, 5 mM CaCl_2_, and 0.02% Brij 35) for 2 h at 37°C. The gel was fixed and stained with 0.5% Coomassie Brilliant Blue R-250 and destained with destaining solution (5% methanol and 1% acetic acid). Areas of protease activity were visualized as clear bands against the blue background on the gel, as a result of the proteolytic digestion of the gelatin in the gel.

### Co-immunoprecipitation assay

Co-immunoprecipitation (co-IP) was performed on the intercellular fluid containing the His-tagged HbSPA and the purified His-T7-tagged PpEPI10 (rPpEPI10) using the T7.tag^®^Affinity Purification Kit (Novagen, Merck Millipore, MA, USA) following the manufacturer’s instructions, with minor modifications. A total of 10 μg of purified rPpEPI10 was pre-incubated with 40 μL of 50% slurry T7.Tag Antibody covalently linked agarose beads for 2 h at 4°C with gentle agitation. The protein/bead complex was pelleted at 2,000 rpm for 10 min followed by three washes with 1x Bind/Wash Buffer to remove unbound protein. Five hundred microliters of the intercellular fluids containing the His-tagged HbSPA were added to the protein/bead complex followed by overnight incubation at 4°C with gentle agitation. The agarose beads were washed three times with 1x Bind/Wash Buffer, followed by centrifugation at 2,000 rpm for 10 min. The precipitated protein complexes were eluted with 40 μL of 1x Elution Buffer and neutralized by adding 6 μL of 1x Neutralization Buffer. The immunocomplexes were subjected to 10% SDS-PAGE and followed by Western blot with HRP conjugated anti-His monoclonal antibody.

### Pathogen infection assay to evaluate the role of HbSPA in plant resistance

The role of HbSPA protein in plant immune response against *P*. *palmivora* was evaluated using droplet inoculation of *P*. *palmivora* zoospore suspension on *N*. *benthamiana* leaves transiently expressing *HbSPA* generated by agroinfiltration. Leaves of 6-week-old *N*. *benthamiana* plants were infiltrated with a mixture of *Agrobacterium* stain C58-C1 carrying pGD-*HbSPA* and *Agrobacterium* stain GV3101 carrying pJL3-*P19* on one half leaf (delineated by the midvein), and on another half *Agrobacterium* stain GV3101 carrying pJL3-*P19* was infiltrated to serve as a control, following the methods described previously [36]. After that, plants were maintained in the laboratory under continuous fluorescent lighting for protein expression. At 36 h post-agroinfiltration, the infiltrated leaves were inoculated with droplets (10-μL) of *P*. *palmivora* zoospores suspension (1x10^4^ spores per mL) on the abaxial surface of the leaf. The plants were kept at high humidity. The diameters of the necrotic lesions were measured at 3 dpi. At least three independent experiments were performed for this assay.

### Statistical analysis

The transcript abundance of *HbSPA* was analyzed using the one-way analysis of variance (ANOVA) according to Tukey’s HSD test using SPSS Statistics 17.0 software. The disease lesion size measurements on *N*. *benthamiana* plants after inoculation with *P*. *palmivora* zoospores were analyzed by comparing the lesion size in the *HbSPA*-expressed half with the lesion size in the control half of each inoculated leaf using the paired t-test with R.

## Results

### Expression of *PpEPI10* and *HbSPA* genes during infection of rubber tree by *P*. *palmivora*

**Fig 1:**
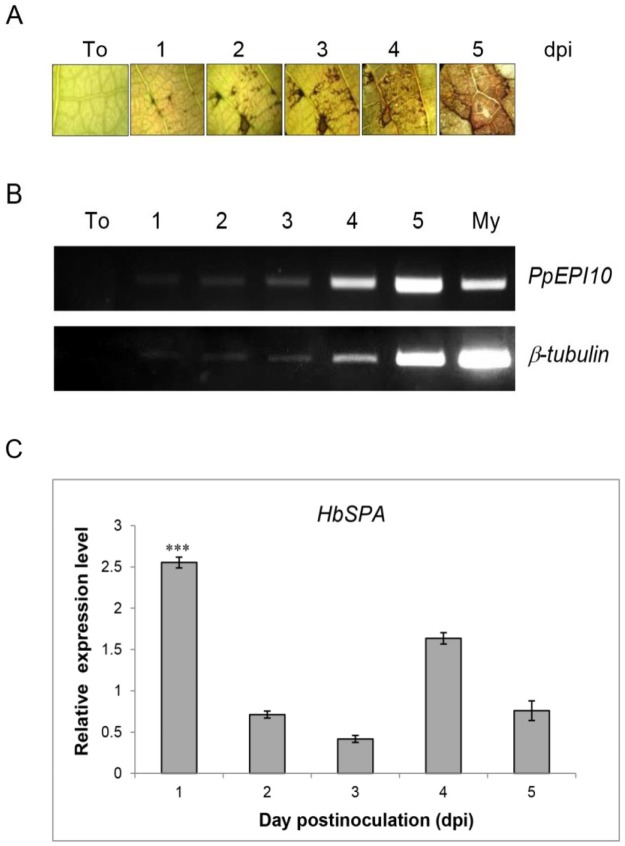
Expression of *PpEPI10* and *HbSPA* genes in rubber tree infected by *P*. *palmivora*. (A) Symptoms observed on noninfected rubber leaves (To), and infected rubber leaves after inoculation with zoospores of *P*. *palmivora* under a light microscope at 1, 2, 3, 4, and 5 days post-inoculation (dpi). (B) Semi-quantitative RT-PCR analysis of *PpEPI10* and *P*. *palmivora β-tubulin* expression during colonization of rubber tree by *P*. *palmivora*. Total RNAs isolated from noninfected leaves (To), infected leaves of rubber tree at 1, 2, 3, 4, or 5 dpi, and *P*. *palmivora* mycelium grown in synthetic medium (My) were used for the analysis. The constitutive *P*. *palmivora β-tubulin* gene was used as a control to determine the relative expression of *PpEPI10*. (C) Real-time RT-PCR analysis of *HbSPA* expression during colonization of rubber tree by *P*. *palmivora*. Total RNA from a time course similar to the one described in (B) was used. The expression of *HbSPA* gene was expressed as a relative transcript fold change to their non-infected controls. The columns and vertical bars represent a mean ± standard errors (SE) of the relative expression levels calculated from the three independent experiments. Three asterisks (***) indicate statistically significant differences in the relative expression levels of *HbSPA* in the infected condition compared to its expression levels in the uninfected condition as a control (P-value < 0.01) determined by paired *t*-test.

To investigate a possible role of HbSPA in defense against *P*. *palmivora* and a possible role of PpEPI10 in *P*. *palmivora* virulence, we determined the expression of *PpEPI10* and *HbSPA* genes during infection of rubber tree by *P*. *palmivora*. We collected the infected leaf tissues across the infection time course, during which increased disease and increased growth of *P*. *palmivora* were observed (Fig 1A). Semi-quantitative RT-PCR analysis was performed to determine the expression of *PpEPI10* gene during colonization of the rubber tree by *P*. *palmivora* and in mycelium cultured *in vitro*. The *PpEPI10* gene was expressed in *P*. *palmivora* across the time course of colonization on rubber tree leaves. Notably, the expression of *PpEPI10* was remarkably induced during infection of rubber tree in contrast to its expression in mycelium grown *in vitro* and relative to the constitutive *P*. *palmivora β-tubulin* gene (Fig 1B), suggesting that *PpEPI10* plays a role in pathogenesis. The expression of *HbSPA* was examined using real-time qRT-PCR analysis. *HbSPA* was significantly up-regulated by 2.5 fold at 1 dpi, abruptly declined thereafter and slightly increased at 4 dpi (1.6 fold), when compared to the relative transcript level of the non-infected control (Fig 1C). The rapid increase in *HbSPA* expression during early infection of rubber tree by *P*. *palmivora* suggests that *HbSPA* plays a potential role in plant defense.

### Functional expression of *Hevea* serine proteases genes in *N*. *benthamiana*

**Fig 2:**
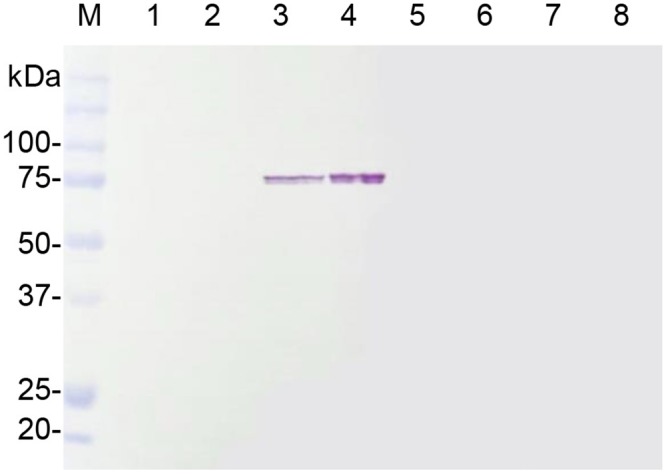
*Agrobacterium*-mediated transient expression of *Hevea* serine protease genes in *N*. *benthamiana*. Total proteins were extracted from agroinfiltrated *N*. *benthamiana* leaves and subjected to SDS-PAGE followed by Western blot with HRP conjugated anti-His monoclonal antibody. Lane M represents the protein standard and the numbers on the left indicate the size of molecular weight markers. Lanes1-8 represent total proteins extracted from *N*. *benthamiana* leaves infiltrated with the infiltration media (Mock, lane 1), *A*. *tumefaciens* GV3101 carrying the pJL3-*P19* (lane 2), *A*. *tumefaciens* C58-C1 carrying the pGD-*HbSPA* (lane 3), a mixture of *A*. *tumefaciens* strains carrying the *pGD-HbSPA* and the pJL3*-P1*9 (lane 4), *A*. *tumefaciens* C58-C1 carrying the *pGD*-*HbSPB* (lane 5), a mixture of *A*. *tumefaciens* strains carrying the pGD-*HbSPB* and the pJL3-*P19* (lane 6), *A*. *tumefaciens* C58-C1 carrying the pGD-*HbSPC* (lane 7), a mixture of *A*. *tumefaciens* strains carrying the pGD-*HbSPC* and the pJL3-*P19* (lane 8), respectively.

In order to express *Hevea* subtilisin-like serine protease genes in *N*. *benthamiana*, the coding sequences of *HbSPA*, *HbSPB*, and *HbSPC* fused to the hexahistidine (His)-tag at the C-terminus were cloned into pGD and transiently expressed in *N*. *benthamiana* leaves by *A*. *tumefaciens* mediated transient expression (agroinfiltration). The expression of HbSPA, HbSPB, and HbSPC proteins was analyzed in total protein extracts of agroinfiltrated *N*. *benthamiana* leaves by Western blot with HRP conjugated anti-His monoclonal antibody. Only *HbSPA* gene was successfully expressed in *N*. *benthamiana* leaves as visualized by Western blot showing a corresponding protein band of approximately 75-kDa (Fig 2, lanes 3 and 4). Moreover, an increase in the expression level of HbSPA protein was observed when co-expressing HbSPA and P19 compared to expressing HbSPA alone, demonstrating that the employment of gene silencing suppressor P19 protein derived from *Tomato bushy stunt virus* (TBSV) to suppress the onset of post-transcriptional gene silencing was able to enhance the expression level of HbSPA in *N*. *benthamiana* (Fig 2, lane 4).

**Fig 3:**
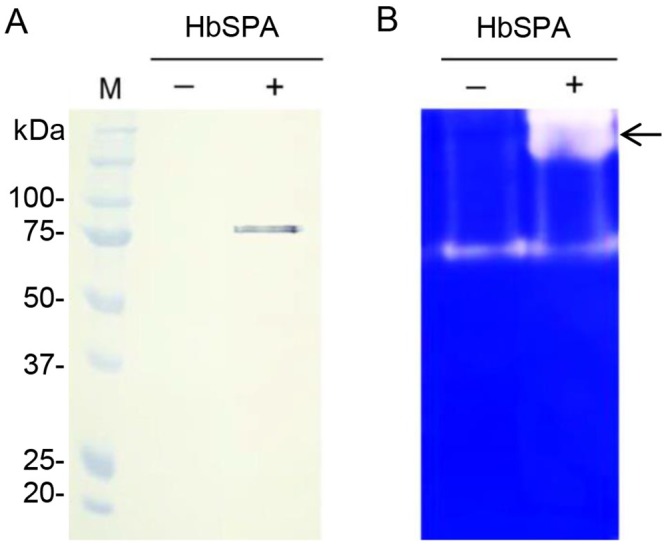
Transient expression of *HbSPA* in *N*. *benthamiana*. Intercellular fluids were isolated from *N*. *benthamiana* leaves infiltrated with *A*. *tumefaciens* GV3101 carrying the pJL3-*P19* as a control (- HbSPA), a mixture of *A*. *tumefaciens* strains carrying pGD-*HbSPA* and pJL3-*P19* (+ HbSPA). (A) Samples were analyzed by SDS-PAGE followed by Western blot with HRP conjugated anti-His monoclonal antibody. Lane M represents the protein standard and the numbers on the left indicate the size of molecular weight markers. (B) Samples were analyzed by zymogram in-gel protease assay. Samples were loaded on a SDS-PAGE containing 0.1% of gelatin. After electrophoresis, proteins were renaturated in the gel and the protease activity was detected after a 2-h incubation required for gelatin degradation. The clear and distinct bands against the blue background on the gel corresponding to the protease activity associated with the presence of the HbSPA protein was indicated by an arrow.

To identify whether the recombinant HbSPA protein was expressed in the apoplast of *N*. *benthamiana*, the intercellular fluids were recovered from the whole leaves of *N*. *benthamiana* plants infiltrated with *A*. *tumefaciens* GV3101 carrying pJL3-*P19* or a mixture of *A*. *tumefaciens* strains carrying pGD-*HbSPA* and pJL3-*P19* using a vacuum infiltration method, and subjected to Western blot analysis. The protein band of HbSPA was detected in samples from leaves co-infiltrated with *A*. *tumefaciens* strains carrying pGD-*HbSPA* and pJL3-*P19*, but not from leaves infiltrated with *A*. *tumefaciens* GV3101 carrying only pJL3-*P19*, indicating that the recombinant HbSPA protein was an extracellular protein (Fig 3A).

To further determine whether the recombinant HbSPA protein was a functional protease, the intercellular fluids isolated from *N*. *benthamiana* leaves infiltrated with *A*. *tumefaciens* GV3101 carrying pJL3-*P19* or a mixture of *A*. *tumefaciens* strains carrying pGD-*HbSPA* and pJL3-*P19* were subjected to zymogram in-gel protease assay. The zymogram gel revealed an additional protease band in the sample containing HbSPA protein, but not in the control, indicating that the recombinant HbSPA protein was a functional extracellular protease (Fig 3B).

### Ectopic expression of *HbSPA* in *N*. *benthamiana* enhances resistance to *P*. *palmivora*

**Fig 4:**
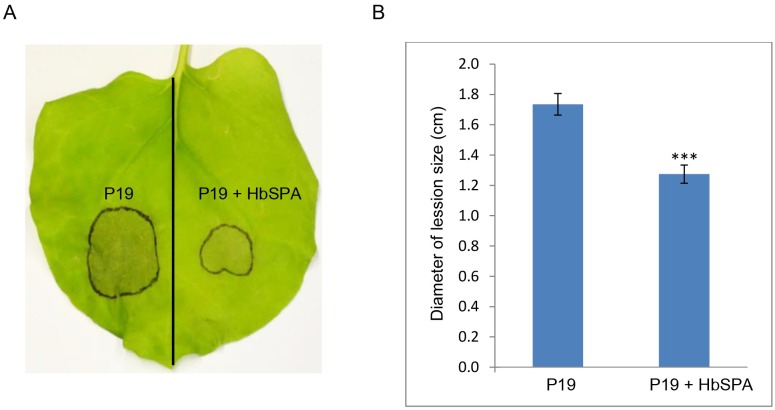
Increased resistance mediated by *HbSPA* expressed in *N*. *benthamiana* to *P*. *palmivora*. (A) Infection symptoms of *P*. *palmivora* on a representative *N*. *benthamiana* leaf with one half transiently co-expressing *HbSPA* and *P19* genes and another half expressing *P19* gene only as a control. The photograph was taken at 3 days post inoculation (dpi). (B) Average lesion size at 3dpi on *N*. *benthamiana* leaf halves expressing *P19* gene only or both *P19* and *HbSPA* following drop inoculation with 100 zoospores of *P*. *palmivora*. The histograms correspond to the mean ± standard errors (SE) of lesion diameter calculated from the three independent experiments (n = 150). Three asterisks (***) indicate statistically significant differences in the lesion diameters derived from *N*. *benthamiana* leaves with one half co-expressing *HbSPA* and *P19* genes in the infected condition compared to another half expressing *P19* gene only in the same condition as a control (P-value < 0.01) determined by paired *t*-test.

To evaluate the effect of *HbSPA*-expressing *N*. *benthamiana* plants against *P*. *palmivora* infection, we expressed HbSPA protein in *N*. *benthamiana* leaves via *Agrabacterium*-mediated transient transformation. *N*. *benthamiana* leaf was separated into two halves delineated by the midvein. The first half was infiltrated with *A*. *tumefaciens* GV3101 carrying pJL3-*P19* and the other half was infiltrated with a mixture of *A*. *tumefaciens* strains carrying pGD-*HbSPA* and pJL3-*P19*. The agroinfiltrated leaves were then inoculated with droplets (10-μL per droplet) containing ~100 zoospores of *P*. *palmivora*. Obvious differences in disease symptoms were observed at 3 dpi. Ectopic expression of HbSPA protein significantly reduced the localized necrotic area (Fig 4). The average lesion size in the halves expressing HbSPA protein (1.27-cm-diameter) was smaller than in the control halves of the same leaves (1.73-cm-diameter) (Fig 4B). This result suggested that *HbSPA* gene plays a significant role in plant resistance against *P*. *palmivora* infection.

### Inhibition of protease activity of HbSPA by PpEPI10

**Fig 5:**
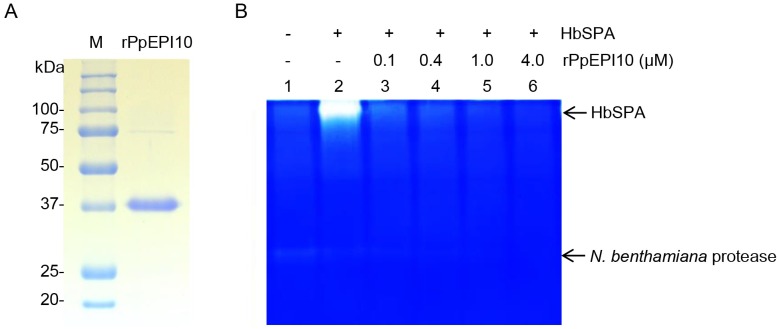
The protease activity of HbSPA is inhibited by rPpEPI10. **(A)** Affinity-purified rPpEPI10 visualized on 12% SDS-PAGE followed by Coomassie Brilliant Blue staining. Lane M represents the protein standard and the numbers on the left indicate the size of molecular weight markers. Lane rPpEPI10 represents the purified rPpEPI10 eluted from the Ni-NTA His-Bind^®^ affinity column. (B) Inhibition of the protease activity of HbSPA by rPpEPI10. The intercellular fluids isolated from *N*. *benthamiana* leaves infiltrated with *A*. *tumefaciens* GV3101 carrying the pJL3-*P19* were incubated with buffer (25 mM HEPES, pH 7.5, 150 mM NaCl, 10% glycerol) (lane 1), and the intercellular fluids isolated from *N*. *benthamiana* leaves infiltrated with a mixture of *A*. *tumefaciens* strains carrying pGD-*HbSPA* and pJL3-*P19* were incubated with buffer (lane 2) or the purified rPpEPI10 at concentrations indicated (lanes 3–6). The remaining protease activity of each sample was determined using zymogram in-gel protease assay. The upper and lower arrows indicate the band location corresponding to the protease activity of HbSPA and a *N*. *benthamiana* protease, respectively.

To determine whether *P*. *palmivora* PpEPI10 targets HbSPA, the recombinant PpEPI10 (rPpEPI10) protein, which is composed of mature PpEPI10 fused with a hexahistidine (His) tag followed by a T7 tag at the N-terminus, was expressed and purified from pET28a-*PpEPI10* in *E*. *coli* BL21 (DE3). The purified rPpEPI10 appeared as a major band of 37 kDa and a minor, barely detectable band of 75 kDa on a SDS-PAGE gel, indicating high purity of rPpEPI10 (Fig 5A).

To investigate the antagonistic activity of rPpEPI10 against HbSPA, the intercellular fluids isolated from agroinfiltrated *N*. *benthamiana* leaves expressing HbSPA protein were incubated in the absence of protease inhibitor (buffer) or the presence of various concentrations of rPpEPI10. The remaining protease activity of HbSPA protein in each incubation sample was detected using the zymogram in-gel protease assay (Bio-RAD, CA, USA). Increasing concentrations of rPpEPI10 exhibited dose-dependent inhibition of the protease activities of HbSPA and a *N*. *benthamiana* protease as these protease bands diminished on the gel after incubation with rPpEPI10 (Fig 5B). This suggests that *PpEPI10* encodes a functional protease inhibitor that inhibits the protease activity of HbSPA.

### Interaction of HbSPA with PpEPI10

**Fig 6:**
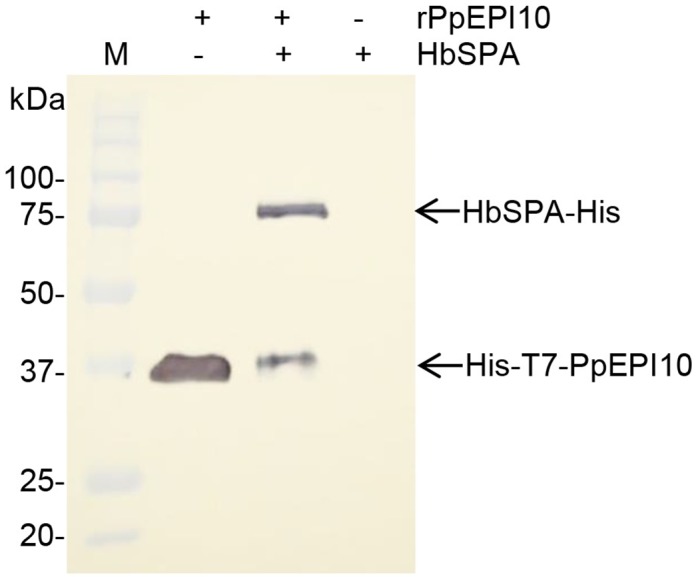
Co-immunoprecipitation of HbSPA and rPpEPI10. The T7 antibody covalently linked agarose beads were incubated with the purified His-T7-tagged PpEPI10 (+ rPpEPI10) or buffer (- rPpEPI10). The mixture was added to buffer (- HbSPA) or the intercellular fluids isolated from *N*. *benthamiana* leaves expressing HbSPA-His protein (+ HbSPA). After washing, the eluates were analyzed by Western blot with HRP conjugated anti-His monoclonal antibody. Lane M represents the protein standard. The numbers on the left indicate the size of molecular weight markers.

To determine whether HbSPA protein is targeted by PpEPI10, co-immunoprecipitation (co-IP) was performed on the intercellular fluids extracted from *N*. *benthamiana* leaves expressing HbSPA-His protein incubated with the purified His-T7-tagged rPpEPI10 or buffer control using the T7 antibody covalently linked agarose beads. The eluted solutions from co-IP were analyzed by Western blot with HRP conjugated anti-His monoclonal antibody. The HbSPA-His protein expressed in the intercellular fluids could only be pulled down in the presence of rPpEPI10 but not in the absence of rPpEPI10 (Fig 6), indicating rPpEPI10 interacted with HbSPA protein which represented a plant protease target of PpEPI10.

## Discussion

Like other plant pathogens, oomycete *Phytophthora* species secrete various effector proteins to suppress host defense responses and promote colonization [40–42]. Elucidation of the functions of effectors and the underlying molecular mechanisms involved in pathogenicity remains challenging. Previously, the *PpEPI10* gene, encoding a Kazal-like extracellular serine protease inhibitor from *P*. *palmivora*, has been implicated in pathogenicity through suppressing plant protease activity in rubber tree [25]. However, the inhibited rubber tree protease(s) was not identified. In this study, we expressed *H*. *brasiliensis* subtilisin-like serine protease genes (*HbSPA*, *HbSPB*, and *HbSPC*) in *N*. *benthamiana* plant using *Agrobacterium*-mediated transient expression to elucidate their possible biological role in plant defense responses. We also examined whether these *Hevea* serine proteases could be targets of PpEPI10. Our results indicated that HbSPA was involved in plant defense responses against *P*. *palmivora*, and targeted by PpEPI10 effector, which is induced and secreted into the plant apoplast from *P*. *palmivora* during infection.

A large number of subtilisin-like serine proteases have been linked to plant immune responses [7,13,23]. Tomato subtilases P69B and P69C are induced and accumulated in the plant extracellular matrix during pathogen attack of tomato [6,19–20]. It was proposed that P69C proteolytically processes an extracellular matrix associated leucine-rich repeat protein (LRP) in viroid-infected tomato to activate plant defense signal transduction processes [22]. In *Arabidopsis*, an extracellular subtilase (SBT3.3) homologous to tomato P69C was found to be a crucial component mediating the pathogen recognition and activating the expression of SA-responsive defensive genes for priming immune responses [23]. Transgenic *Arabidopsis* plants overexpressing *SBT3*.*3* gene showed enhanced resistance to *P*. *syringae* and the oomycete *Hyaloperonospora arabidopsidis* [23]. In cotton (*G*. *babardense*), the expression of *GbSBT1* gene is induced by *V*. *dahliae* infection, jasmonic acid, and ethylene treatments and the *Gb*SBT1 protein interacts with the effector PHB protein secreted from *V*. *dahliae* [24]. Ectopic expression of *GbSBT1* gene in *Arabidopsis* exhibited the enhanced resistance to *F*. *oxysporum* and *V*. *dahliae* [24]. Similarly, the expression of *HbSPA* was found to be increased during infection of rubber tree by *P*. *palmivora*. In addition, *N*. *benthamiana* leaves expressing HbSPA protein were more resistant to *P*. *palmivora* infections, substantiating its value in establishing effective defense mechanisms of the rubber tree. However, the exact biochemical mechanism by which HbSPA protein confers plant defense is unclear and needs to be further elucidated. It may participate in pathogen recognition, immune signaling activation, and/or the proteolytic degradation of pathogen proteins.

In this study, we found the elevated *PpEPI10* gene expression during colonization of the rubber tree by *P*. *palmivora*, which is similar to the expression of *epi1* and *epi10* genes that were previously reported to be highly induced during infection of tomato by *P*. *infestans* [6–7]. We also found that PpEPI10 interacts and inhibits HbSPA via co-immunoprecipitation and zymogram in-gel protease assay. Altogether, these results suggest that PpEPI10 potentially functions as a disease effector that contributes to the pathogenicity of *P*. *palmivora* by targeting plant defensive protease HbSPA. As the expression of *HbSPA* is also induced during infection, the concurrent expression of *PpEPI10* and *HbSPA* during infection and the facts that both encode secreted proteins support the possibility of the direct interaction between PpEPI10 and HbSPA at the infection interface. Moreover, it has been demonstrated that the two Kazal domains of PpEPI10 inhibit and interact with a 95 kDa rubber tree protease [25], implying that PpEPI10 may possibly target multiple rubber tree proteases in the pathogenic process.

The inhibition of plant defensive proteases is one of the multitudinous countermeasures that plant pathogens have developed to shut down host defense mechanisms and colonize plant tissue. For example, *P*. *infestans* secreted two distinct and structurally divergent Kazal-like extracellular protease inhibitors (EPI1 and EPI10) to recognize the same tomato defense-related protease P69B [6–7]. In addition, *P*. *infestans* secretes cystatin-like extracellular protease inhibitors, called EPICs. EPIC1 and EPIC2B were found to bind and inhibit PIP1 and RCR3, two tomato defense papain-like cysteine proteases in the apoplast during infection [43–44]. The inhibition of host proteases by *P*. *infestans*-derived inhibitors may represent a major virulence mechanism in this pathogen. Our finding that the rubber tree defensive protease HbSPA is targeted by *P*. *palmivora* PpEPI10 suggests that a similar virulence mechanism is employed by another *Phytophthora* spp. Plant defense and pathogen counterdefense mediated by interactions of plant proteases and pathogen protease inhibitors may represent a common mechanism of the antagonistic interactions between various *Phytophthora* spp. and their hosts.
